# Size-tunable Synthesis of Silver Nanobelts Using a Polyaniline Derived Polymer as a Template

**DOI:** 10.1038/srep44796

**Published:** 2017-03-20

**Authors:** Sudakar Padmanaban, Minog Kim, Sungho Yoon

**Affiliations:** 1 Department of Bio & Nano Chemistry, Kookmin University, 861-1, Jeongneung-dong, Seongbuk-gu, Seoul, Republic of Korea

## Abstract

Silver nanobelts (AgNBs) have attracted a great interest due to their excellent electrical conductivity and mechanical strength, leading a facile synthesis of these AgNBs in great demand. In here, we are reporting a simple, aqueous phase, size tunable synthesis of smooth surfaced 1D-silver nanobelts using a Polyaniline (PANi) derived polymer at room temperature. The specifically designed PANi polymer, comprising a pendant carboxyl group in the chain, acted as both a reducing agent and template. The resulting Ag nanobelts have more than 10 μm of length, mean width values ranging from 41.1 (11.5) nm to 118.5 (8.8) nm and a mean thickness value of 36.7 (12.5) nm. The UV-Visible spectrum of the AgNBs has shown two Surface Plasmon Resonance peaks at 352 nm and 383 nm.

Silver nanomaterials have been extensively studied in recent years and their applications in various fields such as photochemistry, catalysis, sensors, fabrication of electronic devices, biochemical sensing and imaging have attracted significant attention from the scientific community. Among the other structures of silver nanomaterials, one dimensional (1-D) silver nanowires (AgNWs) and nanobelts (AgNBs) have been of particular interest as they are exhibiting excellent electrical conductivity and mechanical strength^1^,^2^. AgNWs have been employed for numerous applications such as transparent heaters, transparent and flexible electronics, nanocomposites and nanosensors^3^,^4^,^5^,^6^,^7^,^8^. On the other hand, the thermally stable AgNB is also a high aspect-ratio nanomaterial and is flexible in nature. More flexible, ultra-long AgNBs will be more favored for the fabrication of flexible and transparent nanoelectronic devices. In addition, the wide surfaces of the AgNBs are expected to exhibit higher catalytic activities than the AgNWs.

Several methods have been reported for the preparation of AgNBs, including wet chemical^9^,^10^,^11^,^12^,^13^ and electrochemical methods^14^,^15^,^16^,^17^,^18^. However, these procedures need the simultaneous addition of reducing agents and templates at either high or very low temperatures, which makes controlling the size and morphology of the system more complicated. We hypothesized that these complications could be lessened if the number of required reagents were reduced in the synthesis. Thus, combining reducing agent and template or capping agent together to form a simple polymeric motif that could serve as both a template and a reducing agent would be a facile method to produce 1D Ag nanomaterials. In here, we show a polyaniline based polymer with pendant carboxylic groups (Fig. 1) that act both as a template and reducing agent to prepare AgNB from silver nitrate.

## Results and Discussion

The PANi polymer was synthesized as described in the methods section. Solid state ^13^C-NMR of the synthesized PANi polymer confirmed the anthranilic acid and 1,3-propane sultone repeating units to be in 52:48 ratio (Supplementary Fig. S2). The synthesized PANi derivative was then applied for the synthesis of AgNB and the resulting Ag material was analyzed using scanning electron microscopy (SEM). The newly synthesized Ag nanomaterials were found to be long NBs (AgNBs), with mean width values ranging from 41.1 (11.5) nm to 118.5 (8.8) nm and a mean thickness value of 36.7 (12.5) nm. (Fig. 2 and Supplementary Fig. S3). The length of the AgNBs, measured by SEM, were found to be longer than 10 μm (the mean length of AgNB around 14.0 (8.9) μm were observed besides the main bundles of AgNB which are fragments of long AgNBs in the bundles). Transmission electron microscopy (TEM) images of the synthesized AgNBs are shown in Fig. 2c,d and also in Supplementary Fig. S4d; the crooked shape in Fig. 2d, and Supplementary Fig. S4d and the inset in Supplementary Fig. S4d demonstrate the possible flexibility of AgNBs. Focused ion beam (FIB) images (Fig. 2e,f and Supplementary Fig. S5) clearly show that the as-synthesized nanomaterials are AgNBs; the square-shaped cross-section of the 1D AgNBs (Supplementary Fig. S5a) differentiated them from AgNWs (Supplementary Fig. S5b), which exhibit a pentagonal cross-section consisting of five trigonal-shaped crystals^19^,^20^. Furthermore, when comparing the SEM and TEM images of the AgNW and AgNB in Supplementary Fig. S4, AgNBs may be more flexible than AgNWs. Perhaps, the flexibility difference between these two materials might be aroused because of the different lattice arrangements. Consequently, AgNBs can be further applied as conductive material for flexible displays.

The X-ray diffraction (XRD) pattern is well matched with the literature reports (JCPDS No. 04-0783)^12^ and shows that face-centered cubic AgNBs were formed (Fig. 3a). Figure 4a shows the selected area electron diffraction (SAED) pattern, which suggested that the as-formed AgNBs were highly pure. The high-resolution transmission electron microscope (HRTEM) images (Fig. 4b and supplementary Fig. S6) also show that the AgNB is highly crystalline, and free from any dislocations. The HRTEM image in Fig. 4b shown clear fringes with a spacing of ca. 0.198 nm parallel to the edge that corresponds to {020} reflections. Additionally, the fringes with a spacing of ca. 0.204 nm perpendicular to the edge was seen that corresponds to {200} reflections. It suggested that the AgNB may be growing along the <200> direction. The Fast Fourier Transform (FFT) image of the AgNBs, shown in the inset of Fig. 4b, also demonstrates that the growth direction is <200> which is different from the direction <110>, the typical direction of growth that is reported in literature^9^,^10^,^13^,^18^,^21^. Thus, because of this different growth direction, unique applications of the AgNBs could be expected in various fields, including catalysis, photo-electronics, and conductive material fabrications, as AgNBs are known to exhibit diverse properties that depend on the growth faces^22^. Moreover, in the TEM images shown in Supplementary Fig. S4d, an organic layer with a thickness around 6 nm was detected beside the AgNBs. These organic layers were observed beside every AgNB and hence the surfaces of the AgNBs may be considered to be stabilized by the organic PANi layers. To quantify the amount of PANi attached onto the surface of the AgNBs, thermogravimetric analysis (TGA) was performed, and it was found that *ca.* 10 wt% of PANi was coated onto the AgNB surface (Fig. 3b). It indicates that a high amount of the self-capping agent PANi stabilize the surface of the nanomaterial.

Controlling the size of the resulting AgNB is important for tuning its properties and thus the applicability in any field. For example, wider NBs are useful in catalytic applications; on the other hand, thickness control is necessary for scenarios where a thin film of the NB is needed. Hence, to tune the width of the AgNB, the effect of the amount of surfactant used was studied at 30 °C using15 wt%, 30 wt%, 60 wt% and 90 wt% of PANi with respect to the amount of AgNO_3_. Figure 5 shows the SEM images of the AgNBs obtained at 30 °C using different concentrations of the surfactant along with their width histograms. The mean width values are listed in Table 1. When using 15 wt% and 30 wt% of PANi, the mean width values for the AgNBs were calculated to be 41.1 (11.5) nm and 45.6 (13.2) nm (entry 1 and 2 in Table 1). Increasing the PANi amount to 60 wt% and 90 wt% resulted in a gradual increase in the mean width to 61.1 (17.0) nm and 88.6 (29.2) nm, respectively (Entry 3 and 4 in Table 1). These results suggest that, the mean width of AgNBs increases when using higher concentration of surfactant. Subsequently, NB formation was studied at 50 °C, a slightly higher temperature at which larger materials may be more energetically favored as a result of the Ostwald ripening process. As expected, the mean width of the AgNBs was shown to be increased at all concentrations of PANi at 50 °C (Supplementary Fig. S7a-h; entry 5, 6, 7 and 8 in Table 1). However, the large standard deviation values in Table 1 for entry 5 to 8 show that the size distribution was not homogeneous at this increased temperature. Therefore, for a homogeneous size distribution, the reaction was performed at 5 °C using 15 wt%, 30 wt%, 60 wt% and 90 wt% of PANi. At lower temperatures, the reaction was too slow to generate nanobelt upon using 15 wt% and 30 wt% of PANi (Table 1, entry 9 and 10). Interestingly, with 60 wt% of PANi, the AgNB with a mean width of 55.1 (14.8) nm was formed even at 5 °C (Supplementary Fig. S8a & b; Entry 11, Table 1). Further increase of PANi to 90 wt% resulted in the formation of uniformly distributed AgNB with the mean width value, averaging around 118.5 (8.8) nm (Supplementary Fig. S8c & d). Concisely, increasing the amount of PANi at lower temperatures resulted in a near-homogeneous distribution in the width of the AgNB. On the other hand, increasing the PANi amount at 50 °C resulted in a random size distribution and belts with increased mean width values.

A PANi derivative without carboxyl groups in the polymeric back-bone was constructed to investigate whether the carboxyl group can act as a capping agent for the formed Ag seeds to produce AgNBs (Supplementary Fig. S9a). However, the SEM analysis of the resulting precipitate showed that only Ag nanoparticles were formed upon treating 30 wt% and 60 wt% of this PANi derivative with AgNO_3_ at 30 °C (Supplementary Fig. S9b & c). Therefore, it could be proposed that the carboxylate group in the polymeric chain may coordinate locally to the Ag nanocrystal that is formed from the reduction of the Ag^+^ ion (Supplementary Fig. S10). During this process, the crystal face with the highest surface energy may fit best to the local geometry of the carboxylic group within the PANi polymer chain and is expected to be stabilized by combining with the conducting PANi polymer^7^,^23^. As the absolute value of this capping energy is larger for a specific crystal face that is combined with the conductive polymer, the coordinated Ag seeds may combine in a belt shape with each other through (200) planes which is a different crystal face that results in a minimum absolute value of the capping energy^11^,^22^,^24^. Thus, the Ag seeds might be formed and grown on the PANi, and the carboxyl groups force the AgNBs to grow in <200> direction (Supplementary Fig. S10).

The UV-Visible (UV-Vis) spectrum of the AgNBs (Supplementary Fig. S11) shows two surface plasmon resonance peaks at 352 and 383 nm. It has been reported that the silver nanobars with the aspect ratio of 2 were shown to have two resonance peaks at 410 nm and at 450 nm which correspond to transverse resonance peak and longitudinal resonance peak respectively^25^. Similarly, the AgNBs presented in this work is expected to exhibit the transverse resonance and longitudinal resonance peaks in the UV-Vis spectrum since it has similar width and thickness values as indicated by the FIB images. Thus, the peak at 383 nm might be aroused as a result of the transverse oscillation of electrons across the thickness of the belts and the peak at 352 nm might have aroused due to the longitudinal oscillation of electrons along their width direction^18^,^26^,^27^,^28^.

## Conclusions

In conclusion, AgNBs, one of the most important morphologies of Ag nanomaterials, were prepared using a specifically designed polymer, PANi, which could act as both a reducing agent and as a template for stabilizing the reduced Ag nanomaterials to form smooth surfaced AgNBs. Furthermore, to the best of our knowledge, for the first time, the specially designed carboxyl-containing PANi derivative was shown to be capable of forming ultra-long, flexible AgNBs from AgNO_3_ in an aqueous medium at room temperature. The width of the AgNBs could be controlled by varying the amount of PANi. Moreover, distinctive catalytic properties are expected for the newly synthesized AgNBs because of their unusual growth face (200). Currently we are evaluating the potential catalytic applications of the AgNB.

## Methods

### Materials

All reagents were purchased from commercial sources and used as received without further purification. Aniline hydrochloride (97%), 2-aminobenzoic acid (99%), 2-aminophenol (97%), 1,3-phenylenediamine (99+%), and 1,3-propane sultone (97%) were bought from Sigma-Aldrich. Ammonium persulfate (98%), and AgNO_3_ (99%) were bought from Acros Organics. Hydrochloric solution was purchased from Duksan Pure Chemicals Co., Ltd., Korea.

### Experimental Procedures

**Figure 1:**
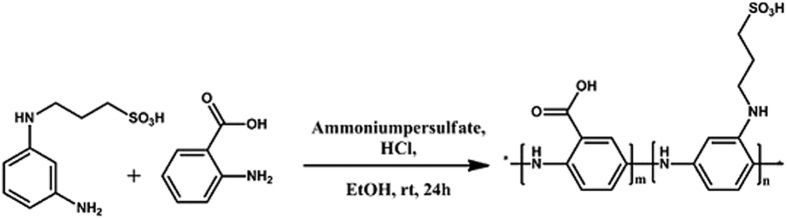
Schematic representation for the synthesis of PANi-derivative comprising carboxylic group and sulfonic acid groups.

To test our hypothesis, we designed a polyaniline (PANi)-based conductive polymer in which carboxyl groups were introduced to coordinate to the Ag^+^ ion and sequentially the PANi motif is expected to convert the Ag^+^ ions into Ag. By doing so, we anticipated the Ag nanomaterials to grow through PANi polymer, so called template. In addition, to perform the reaction in water medium, sulfonic acid groups were also introduced into the PANi motif to improve the water solubility (Fig. 1). The synthesis of the monomer N-(1′,3′-phenylenediamino)−3-propane sulfonate, PANi polymer and the AgNB are given below.

### Synthesis of N-(1′,3′-phenylenediamino)−3-propane sulfonate

To a solution of *m*-phenylenediamine (2.71 g, 25.1 mmol) in tetrahydrofuran (50.0 mL) was added 1,3-propane sultone (3.06 g, 25.1 mmol), and the mixture was refluxed and agitated for 24 h under N_2_ atmosphere (Supplementary Fig. S1). The reaction mixture was cooled to room temperature and the resultant precipitate, collected on a glass filter, was washed with a mixture of 500 mL of THF:methylene chloride 1:1 (v/v), and dried under vacuum to obtain a bluish-gray powder (4.98 g, 87% yield).

^1^H NMR (400 MHz, DMSO-*d*_6_, δ) 7.12 (t, J = 8.0, 1 H), 6.64–6.39 (m, 3 H), 3.12 (t, J = 6.8, 2 H), 2.56 (t, J = 7.2, 2 H), 1.96–1.77 (m, 2 H); IR (KBr): *ν* = 3359(s), 2944(s), 2646(m), 1597(s), 1531(m), 1500(s), 1473(m), 1340(m), 1279(m), 1236(s), 1205(s), 1142(s), 1036(s), 991(w), 868(w), 837(w), 793(w), 750(w), 687(m), 598(s), 565(m), 523(m), 480(w), 451(m), 426 cm^−1^ (w); ESI-MS *m/z* (%): calculated for C_9_H_14_N_2_O_3_S, 230.07, found [M + H]^+^, 231.10.

### Synthesis of Poly[anthranilic acid]_0.5_-[N-(1′,3′-phenylenediamino)−3-propane sulfonate]_0.5_

Anthranilic acid (3.43 g, 25.01 mmol) and *N*-(1′,3′-phenylenediamino)−3-propane sulfonate (5.75 g, 25.00 mmol) were dissolved in a mixture containing 300 mL of 0.2 M HCl solution and 100 mL of ethanol. Ammonium persulfate (APS, 14.21 g, 62.2 mmol), dissolved in 200 mL of 0.2 M HCl solution, was then added to the above solution over 10 minutes, and the mixture was stirred for 24 h. After 24 h, 3.6 L of acetone was added to the solution to obtain a PANi polymer precipitate, which was centrifuged at 4000 rpm for 1 h to separate the precipitate. The precipitate was washed three times with a mixed solution of acetone/0.2 M HCl (6:1 v/v), and dried under vacuum to obtain 6.12 g of poly[anthranilic acid]_0.5_-[*N*-(1′,3′-phenylenediamino)−3-propane sulfonate]_0.5_ (PANi, 66.4% yield). The compositional ratio of the two repeating units in the obtained PANi was confirmed to be 52:48 using Solid State ^13^C-NMR spectroscopy (Supplementary Fig. S2).

### Synthesis of AgNBs

**Figure 2:**
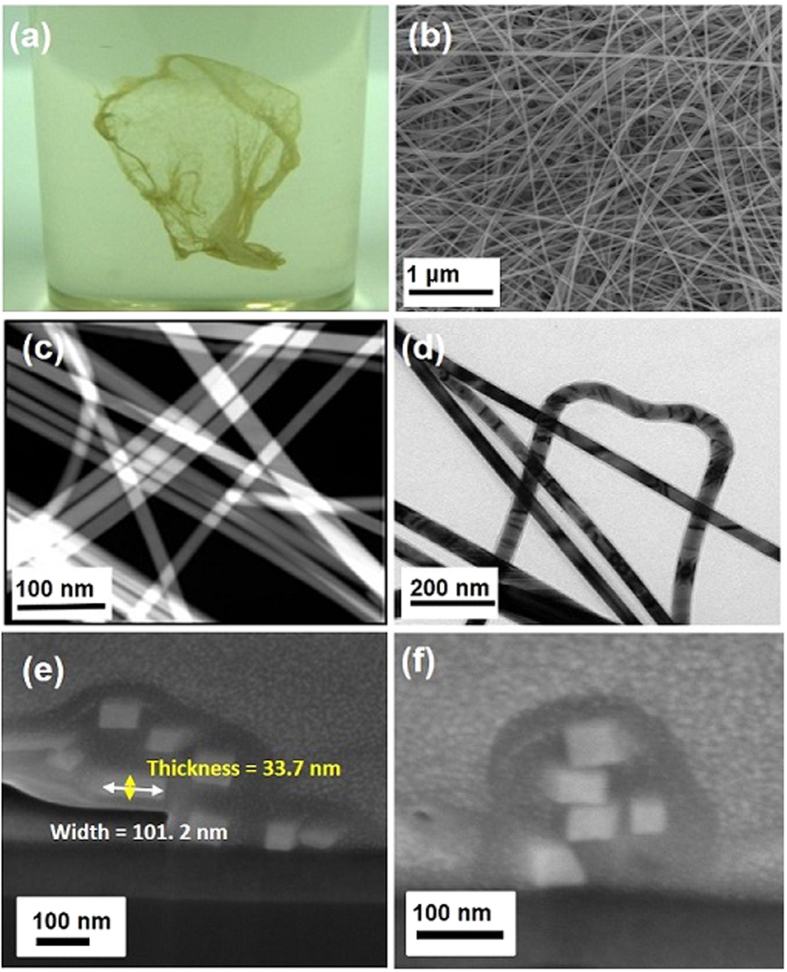
(**a**) Photograph of AgNB synthesized using 15 wt% of PANi at 30 °C in water (**b**) SEM image of AgNB synthesized using 15 wt% of PANi at 30 °C, (**c**) TEM image of AgNB synthesized using 15 wt% of PANi at 30 °C, (**d**) TEM image of AgNB synthesized using 60 wt% of PANi at 30 °C, (**e**) FIB image of AgNB synthesized using 15 wt% of PANi at 30 °C, (**f**) FIB image of AgNB synthesized using 30 wt% of PANi at 30 °C.

**Figure 3:**
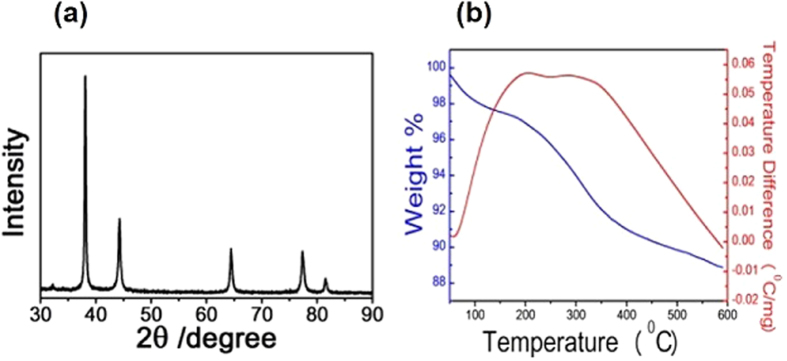
(**a**) XRD data and (**b**) TGA data of AgNB synthesized using 15 wt% of PANi at 30 °C.

**Figure 4:**
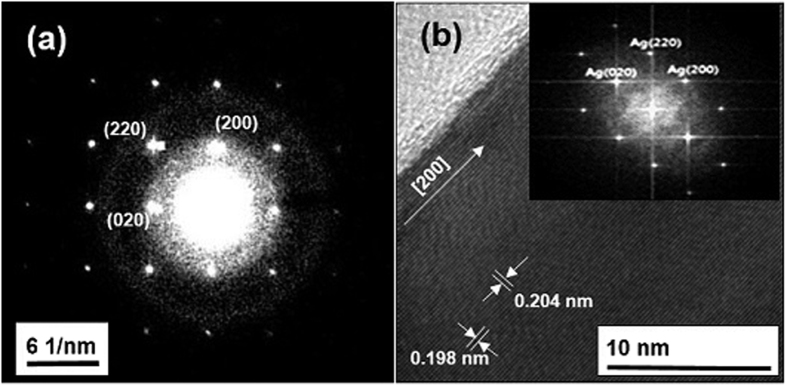
(**a**) SAED pattern, (**b**) HRTEM image; (inset) FFT image of AgNB synthesized using 15 wt% of PANi at 30 °C.

**Figure 5:**
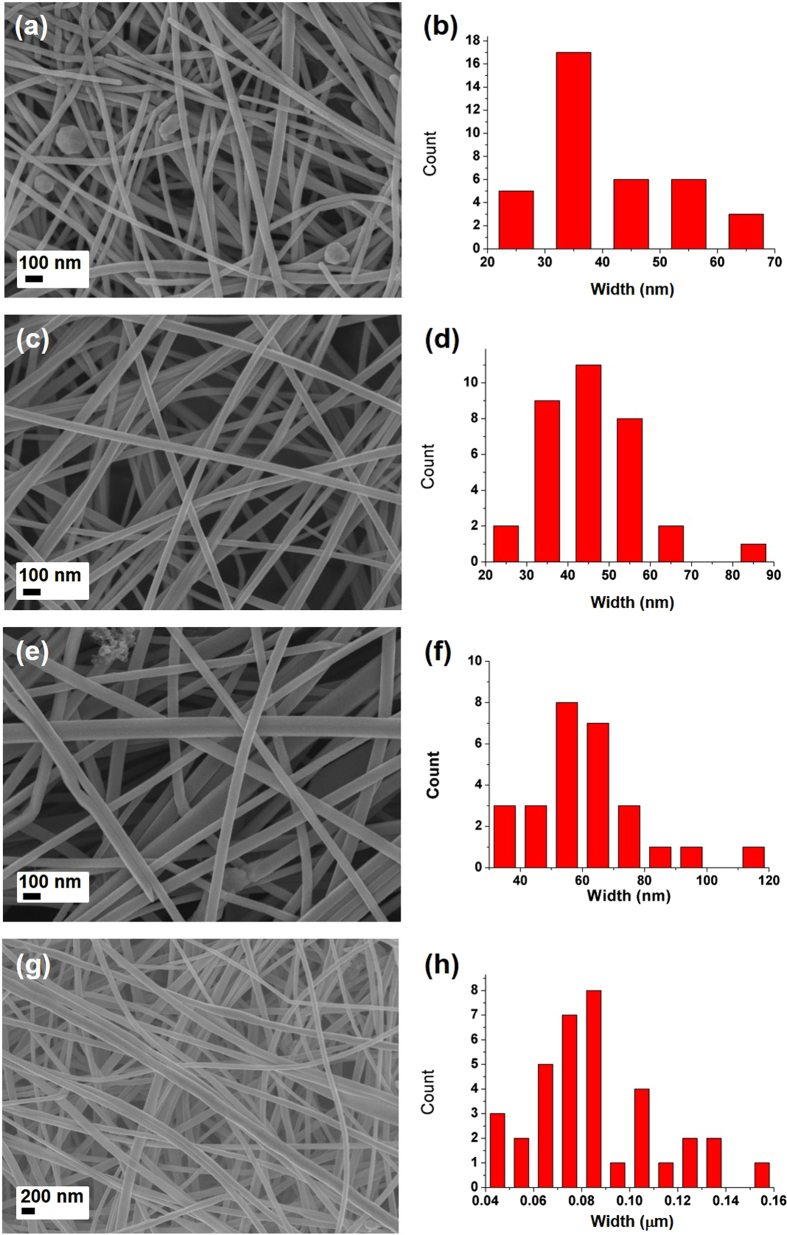
SEM images and the width histograms of AgNBs prepared at 30 °C using 15 wt% (**a** & **b**), 30 wt% (**c** & **d**), 60 wt% (**e** & **f**) and 90 wt% (**g** & **h**) of PANi with respect to the amount of AgNO_3_.

In an initial trial, AgNO_3_ was treated with an aqueous solution of as-prepared PANi polymer at room temperature. The resulting dark green solution was kept at room temperature for 7 days; a jellyfish-shaped film was subsequently obtained in the reaction medium (Fig. 2a). The film was isolated and washed several times with distilled water.

In a typical procedure, 0.025 g of the conductive polymer poly[anthranilic acid]_0.5_-[*N*-(1′,3′-phenylenediamino)−3-propane sulfonate]_0.5_ and 0.170 g of AgNO_3_ (1.00 mmol) were dispersed in 50.0 mL of distilled water and allowed to stand at room temperature for 7 days. The AgNB mass that settled at the bottom was filtered using a filter paper, washed with 50 mL of distilled water, and dried under vacuum for 12 h to obtain 26.2 mg of pure AgNBs.

The approximate length values and the mean width of the AgNBs were measured using SEM. The thickness of the AgNB was measured using Focused Ion Beam (FIB) imaging technique.

### Characterization

Ultraviolet–visible (UV-Vis) spectra were recorded using a UV-Vis spectrometer (X-ma2000, Human Corp., Korea) in the range 300–800 nm. Scanning electron microscope (SEM) images were recorded using a Jeol JSM-7610F, FE-SEM instrument. Transmission electron microscope (TEM) analysis was performed on a Tecnai F30 S-Twin field-emission transmission electron microscope operating at 300 kV. Focused ion beam images were measured using FEI (Nova 600 NanoLab) microscope. Thermogravimetric analysis (TGA) was performed under a nitrogen atmosphere using a 2960 Simultaneous DSC-TGA instrument with a heating rate of 10 °C/min from 25 °C to 600 °C. Powder X-ray diffraction spectra were measured using a Shimadzu XRD-6000 instrument. ^1^H and ^13^C NMR spectra of the products were measured with a Bruker Ascend 400 MHz spectrometer.

## Additional Information

**How to cite this article**: Padmanaban, S. *et al*. Size-tunable Synthesis of Silver Nanobelts Using a Polyaniline Derived Polymer as a Template. *Sci. Rep.* **7**, 44796; doi: 10.1038/srep44796 (2017).

**Publisher's note:** Springer Nature remains neutral with regard to jurisdictional claims in published maps and institutional affiliations.

## Supplementary Material

Supplementary Information

## Figures and Tables

**Table 1:** Effect of temperature and surfactant concentration on the width of the AgNB.

Entry	Temperature	PANi Concentration	Mean width (SD)
1	30 °C	15 wt%	41.1 (11.5) nm
2	30 °C	30 wt%	45.6 (13.2) nm
3	30 °C	60 wt%	61.1 (17.0) nm
4	30 °C	90 wt%	88.6 (29.2) nm
5	50 °C	15 wt%	62.8 (39.5) nm
6	50 °C	30 wt%	67.4 (30.7) nm
7	50 °C	60 wt%	74.5 (36.8) nm
8	50 °C	90 wt%	88.5 (100.6) nm
9	5 °C	15 wt%	Belt did not form
10	5 °C	30 wt%	Belt did not form
11	5 °C	60 wt%	55.1 (14.8) nm
12	5 °C	90 wt%	118.5 (8.8) nm
