# Supplementary material for: Size-tunable Synthesis of Silver Nanobelts Using a Polyaniline Derived Polymer as a Template
Source: Sci Rep. 2017 Mar 20;7:44796. doi: 10.1038/srep44796 (PMC5357892; doi:10.1038/srep44796)
Supplement: Supplementary Information [file srep44796-s1.doc]

Supporting Information For

Size-tunable Synthesis of Silver Nanobelts Using a Polyaniline Derived Polymer as a Template

Sudakar Padmanaban, Minog Kim and Sungho Yoon*

Department of Bio & Nano Chemistry,

Kookmin University, 861-1, Jeongneung-dong, Seongbuk-gu, Seoul, Republic of Korea.
E-mail: yoona@kookmin.ac.kr

**1. Experimental materials**

All reagents were purchased from commercial sources and used as received without further purification. The silver nanowire was synthesized by following a published procedure.^[S1]^

**2. Experimental Methods:**

**2.1. Synthesis of N-(1', 3'-phenylenediamino)-3-propane sulfonate**

**Fig. S1.** ^1^H-NMR spectrum of *N*-(1', 3'-phenylenediamino)-3-propane sulfonate.


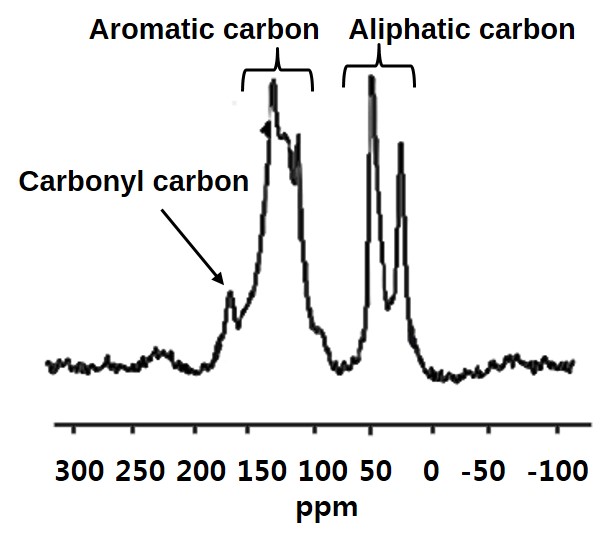
**2.2. Synthesis of Poly[anthranilic acid]0.5-[N-(1',3'-phenylenediamino)-3-propane sulfonate]0.5 (PANi)**

**Fig. S2.** Solid-state ^13^C-NMR spectrum of poly[anthranilic acid]_0.5_-[*N*-(1',3'-phenylenediamino)-3-propane sulfonate]_0.5_

**2.3. Synthesis of Poly[ANi]_0.5_-[N-(1',3'-phenylenediamino)-3-propane sulfonate]_0.5_**

4.72 g of poly[ANi]_0.5_-[*N*-(1',3'-phenylenediamino)-3-propane sulfonate]_0.5_ was prepared as described in Section 2.2 using 3.24 g of aniline hydrochloride instead of anthranilic acid.

**2.4. Reaction of AgNO_3_ with PANi that has no –COOH group in the backbone.**

0.050 g of the conductive polymer poly[ani]_0.5_-[*N*-(1',3'-phenylenediamino)-3-propane sulfonate]_0.5_ and 0.170 g of AgNO_3_ (1.00 mmol) were dispersed in 50.0 mL of distilled water and allowed to stand at room temperature for 7 days. The Ag material that settled at the bottom was filtered using a filter paper, washed with 50 mL of distilled water and then analyzed with SEM.

**3.1 Comparison of AgNBs and AgNWs.**

**
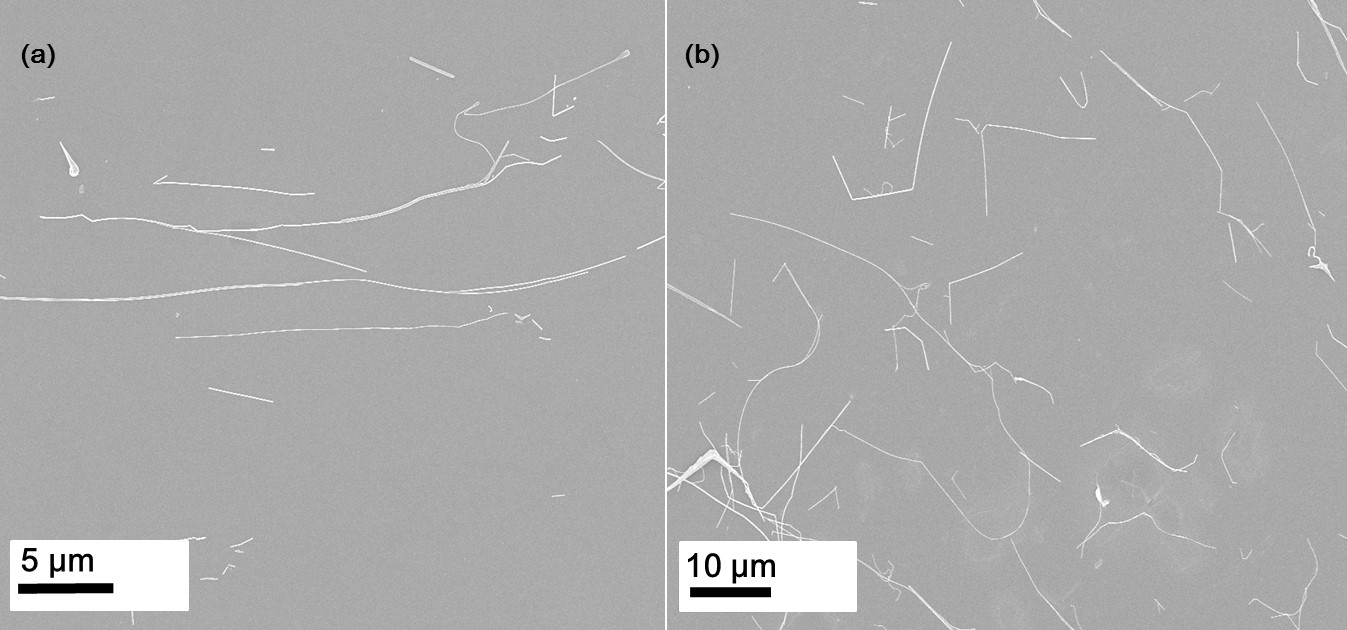
**

**Fig S3.** Lower magnification SEM images of the AgNB showing belts with >10 μm in length. (a) SEM image of AgNB formed at 30 °C using 15 wt% of PANi; (b) SEM image of AgNB formed at 5 °C using 60 wt % of PANi.

**
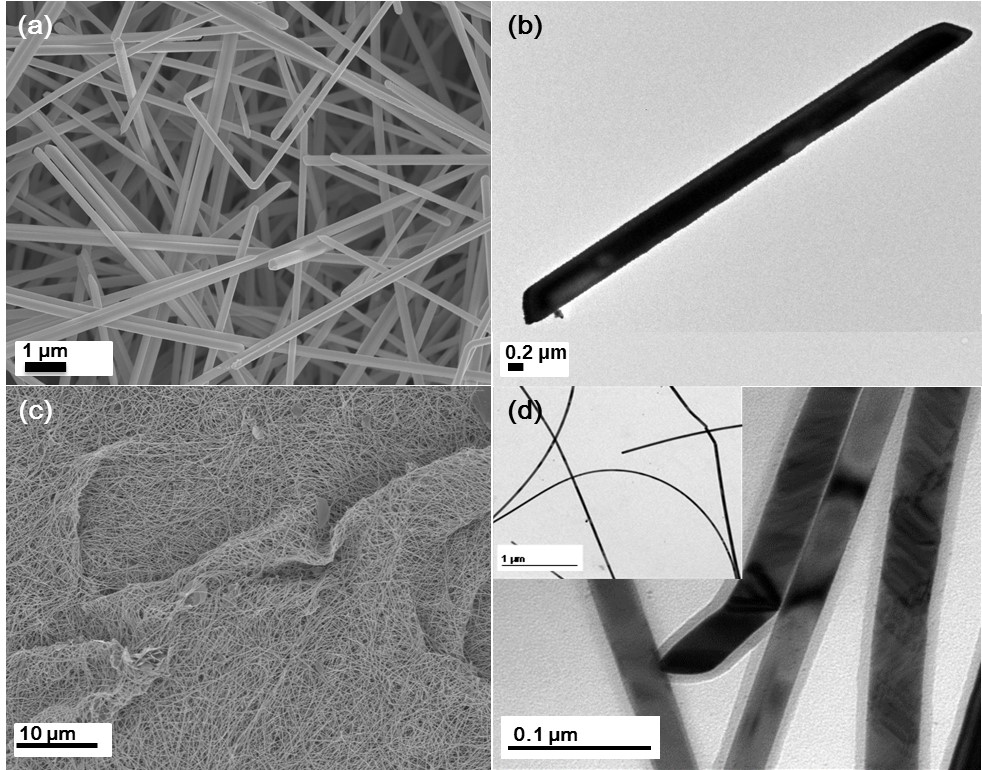
**

**Fig. S4.** (a) SEM image, (b) TEM image of the synthesized AgNW; (c) SEM image; (d) TEM image of AgNB (from entry 2 in Table 1 in the main text); Inset: TEM image of the AgNB at lower resolution that shows the flexibility of the AgNBs.


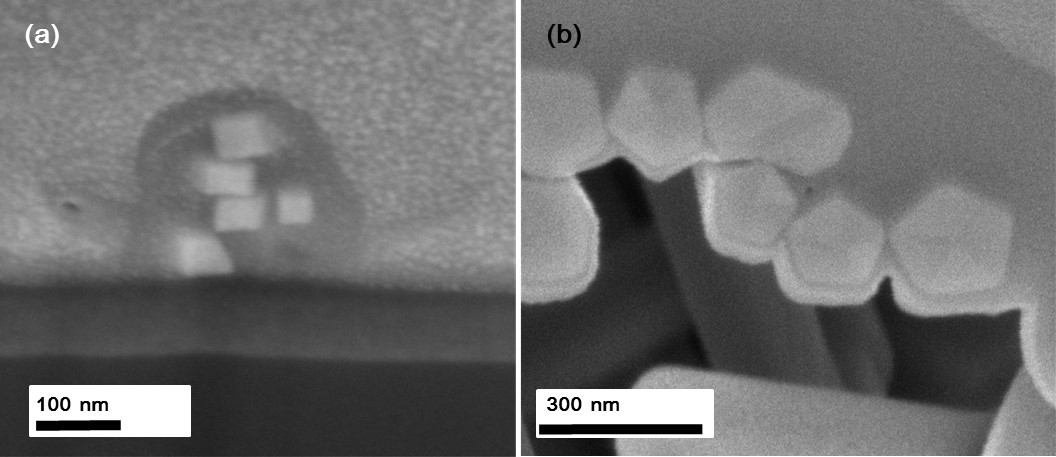


**Fig. S5.** FIB image of 1D Ag nanomaterials: (a) AgNB synthesized using 30 wt% of PANi at 30 °C and (b) FIB image of synthesized AgNWs.


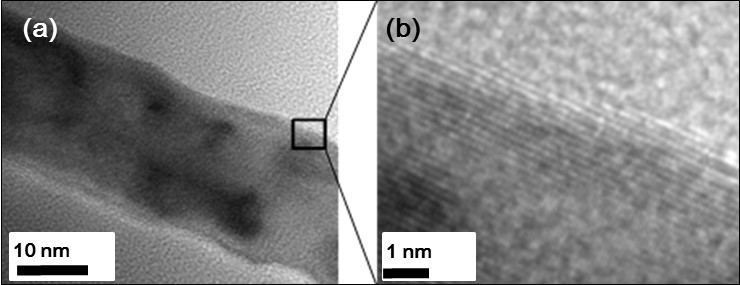


**Figure S6. (a)** TEM image of the AgNB synthesized using 90 wt % of PANi at 5°C. (b) HRTEM image of the same AgNB that shows the crystallinity of the nanobelt.

**3.2. Effect of amount of PANi on as-synthesized AgNBs at different temperatures.**

**
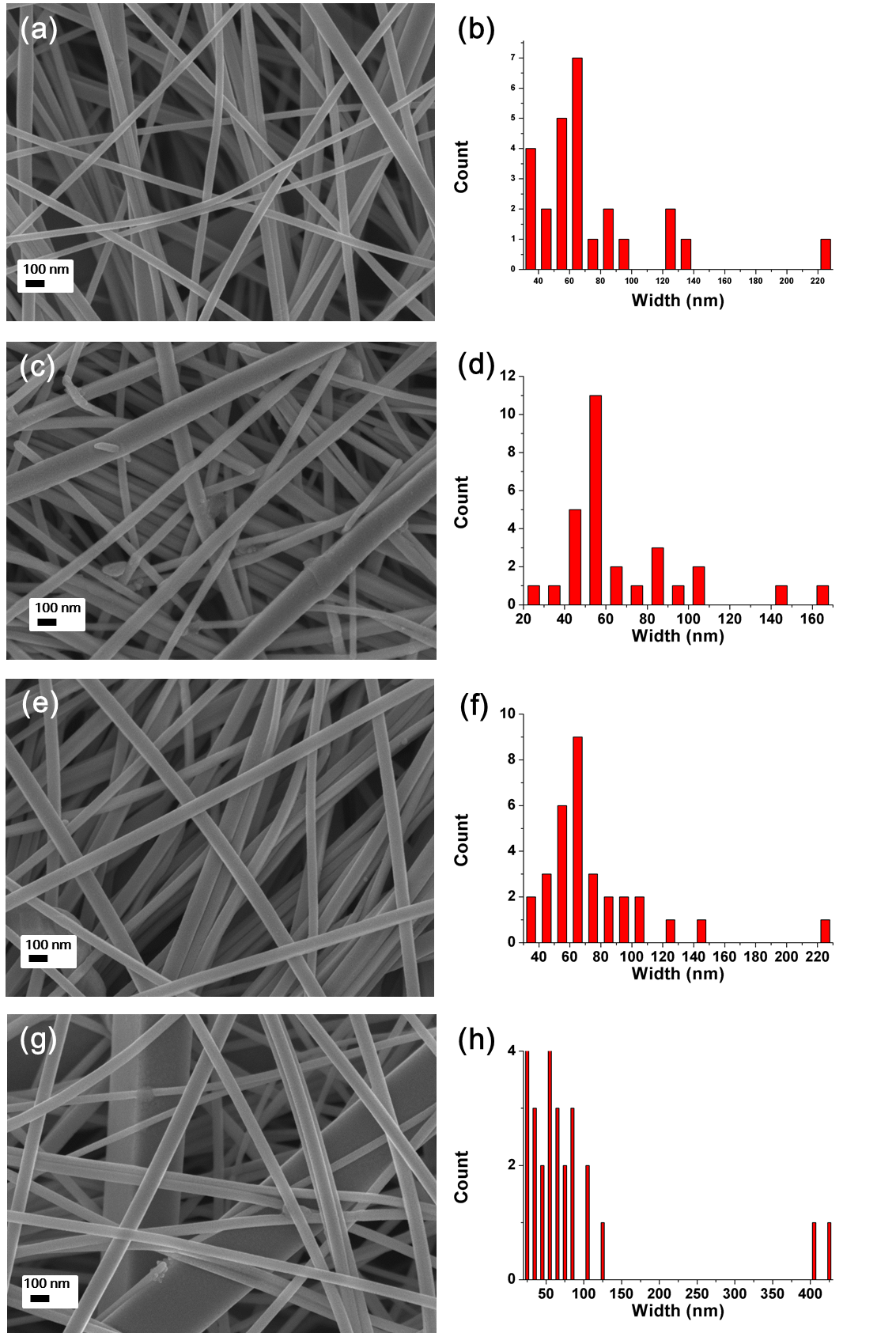
**

**Fig. S7.** SEM images and the width histograms of AgNBs prepared at 50 °C using 15 wt% (a & b), 30 wt% (c & d), 60 wt% (e & f) and 90 wt% (g & h) of PANi with respect to the amount of AgNO_3_.

**
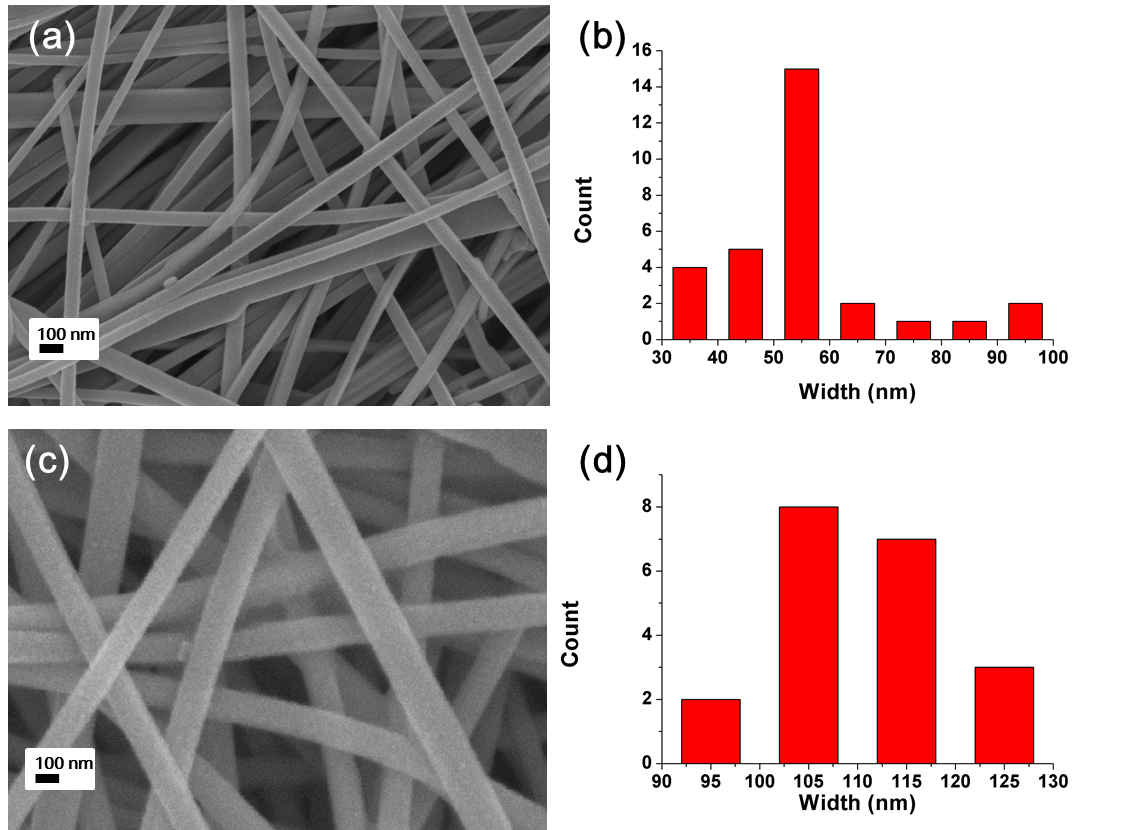
**

**Fig. S8.** SEM images and the width histograms of AgNBs prepared at 5 °C using 60 wt% (a & b) and 90 wt% (c & d) of PANi with respect to the amount of AgNO_3_.

**4.1. PANi derivative without carboxyl groups in the polymeric back-bone:**

**
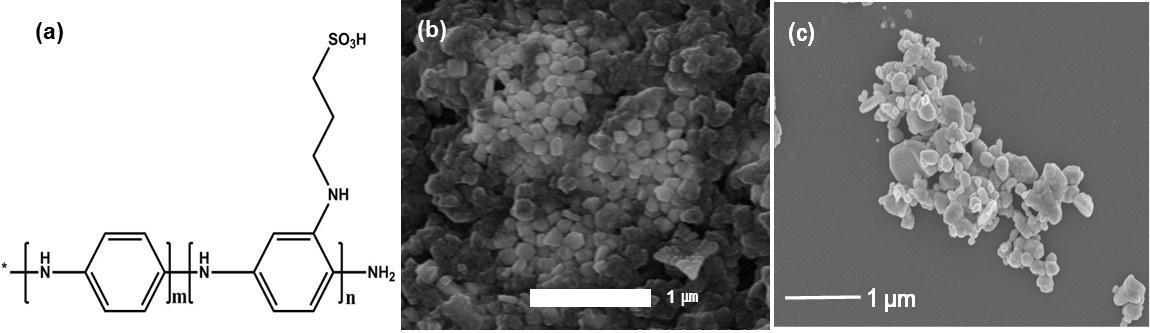
**

**Fig. S9.** (a) PANi derivate without a carboxyl group and SEM image of the resulting Ag Particles using (b) 30 wt% and (c) 90 wt% of surfactant at 30 °C.

**4.2. Plausible Growth Mechanism for the formation AgNB:**

**
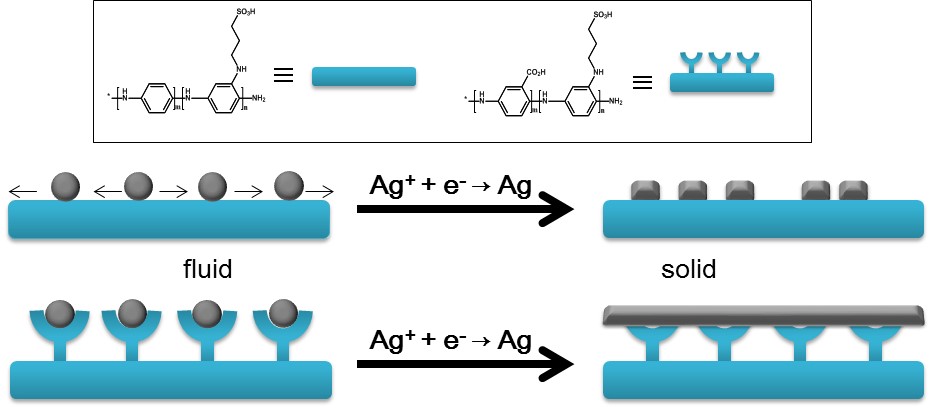
**

**Fig. S10.** Proposed growth mechanism for Ag nanomaterials on PANi Derivatives.

**4.3. UV-Visible spectrum of as-prepared AgNB:**

**Fig. S11.** UV-Visible spectrum of as-prepared AgNB (from entry 1 in Table 1 in the main manuscript) in deionized water.

**Reference.**

[S1]. B. Wiley, Y. Sun, Y. Xia, *Langmuir* **2005**, *21*, 8077.
